# Testing the Effectiveness of a Gamified Emotional Cognitive Bias Modification Task as an Intervention for Low Mood: Randomized Controlled Trial

**DOI:** 10.2196/65103

**Published:** 2025-05-01

**Authors:** Rumeysa Kuruoğlu, Angela Attwood, Ian Penton-Voak

**Affiliations:** 1 School of Psychological Science, University of Bristol, 5 Priory Rd, Bristol, BS8 1TU, United Kingdom, 44 1173746633; 2 NIHR Biomedical Research Centre, University Hospitals Bristol and Weston NHS Foundation Trust, Bristol, United Kingdom

**Keywords:** low mood, mood, depression, depressive, mental health, cognitive bias modification, perceptions, gamification, emotion recognition bias, emotional facial expressions, emotions, facial, expression, biases, mental health

## Abstract

**Background:**

Emotion recognition bias in depression is well-documented and is proposed to play a causal role in depression. A cognitive bias modification (CBM) intervention targeting the bias in emotional expression perception was developed, but despite robust training effects on emotion perception, the effect on mood was unreliable and weak. We propose a new gamified cognitive bias modification (GCBM) to address potential limitations that may attenuate therapeutic effects.

**Objective:**

This study aimed to investigate the effectiveness of a single session of GCBM on emotion perception and to assess whether the gamified version of the task would produce the same robust training effects on the interpretation of emotional expressions as the original CBM. The second aim was to compare the effectiveness of a single session of CBM training, CBM control (no training), and GCBM training on immediate mood.

**Methods:**

We report a between-subjects fully automated and web-based experimental study that recruited participants via the web from the general population (N=916). We tested the effectiveness of GCBM in changing participants’ responses to ambiguous facial expressions. The primary outcome was emotion recognition bias, measured as the increased identification of happy faces. We also compared the effects of a single session of GCBM training (n=397), CBM training (n=400), and CBM control (n=119) conditions on immediate mood, measured using the Immediate Mood Scaler.

**Results:**

Results showed that participants in the GCBM intervention condition classified more ambiguous faces as “happy” after the training compared with controls, indicating an increased perception of happiness in ambiguous faces (B=1.57, *P*<.001). There was also evidence that GCBM training produced more positive changes in immediate mood compared with the CBM control condition (B=−3.64, *P*=.003) and compared with the CBM training condition (B=1.69, *P*=.048).

**Conclusions:**

GCBM demonstrated promising results in changing participants’ emotion recognition bias to ambiguous facial expressions and enhance the immediate mood compared with both CBM and control conditions. These results suggest that GCBM holds promise to be a better alternative to CBM for addressing mood-related cognitive biases. Further exploration of GCBM’s long-term effects on mood and its clinical application is needed.

## Introduction

Depression is associated with a negative bias in the interpretation of facial emotional expressions [1,2]. This negative bias has been proposed to play an important role in the onset and maintenance of depression, as successful pharmacological interventions have been found to be associated with the reduction of negative biases [3]. It was previously thought that treating depression would lead to improvements in emotion recognition bias. However, there is evidence from psychopharmacological studies of antidepressant action that suggests that changes in emotion recognition biases precede and predict later mood improvement [3]. This finding implies that altering negative biases through psychological interventions may be an effective way to improve the mood of individuals with depression [3,4]. Consequently, cognitive bias modification (CBM) interventions that target this negative bias may reduce depressive symptoms [4].

A CBM intervention was developed to aim at changing negative biases in the perception of emotional facial expressions [4]. This CBM technique shifts participants’ responses toward more positive interpretations of ambiguous facial expressions [4]. However, the improvements in mood that are predicted to follow a reduction in negative bias by the neurocognitive models of depression have been weak and unreliable [5-8]. We conducted a meta-analysis of 6 CBM training studies using the same intervention to assess the overall effectiveness of this CBM technique on mood outcomes [9]. The meta-analytic results showed that the effectiveness of the CBM on mood (ie, reducing overall depressive symptoms) was consistent with neurocognitive models of depression; indirect effects of training on improved mood were mediated by decreased negative emotional bias. However, the direct effects of training in the mediation model were in the opposite direction, with allocation to the active training condition leading to lower mood in comparison with the control condition. This was contrary to our predictions and indicated that some aspects of the active training condition were having a negative effect on mood. Overall, the indirect effects (in the therapeutic direction) and direct effects (in the nontherapeutic direction) largely canceled each other; resulting in a weak overall effect of CBM training on mood [9].

In the CBM studies reviewed in our meta-analysis, participants were given negative (ie, “incorrect”) and positive (ie, “correct”) text-based feedback to shift the perception of ambiguous faces from sad to happy (ie, reduce a negative cognitive bias [9]. In the CBM, participants in the training condition receive more negative feedback (ie, are told that they are incorrect) as the mechanism to change their perception of facial emotion from negative (sad) to positive (happy) judgments. Participants in the control conditions receive less negative feedback than those in the active training condition, as the CBM technique does not aim to change their responses from baseline. Based on the meta-analysis results, we speculated that the relatively frequent negative (ie, “incorrect”) feedback in the task might elicit the negative effect of mood associated with the training condition revealed in the meta-analysis. Research suggests that continuous negative feedback decreases positive emotional experiences, and can trigger self-esteem problems and induce negative emotions [10,11]. Furthermore, receiving negative feedback elicits negative emotions, which can reduce the acceptance and use of future feedback [12]. In contrast, positive feedback reduces social anxiety [13] and prevents or attenuates depression [14].

These reported effects of negative feedback suggest that removing or changing the delivery of negative feedback may improve the effectiveness of this CBM technique on mood-related outcomes. However, in order to change emotion recognition bias, some form of feedback is necessary to drive the modification as this “corrects” the response in the intended direction. However, such feedback should be delivered in a constructive way. The education literature points to the importance of constructive feedback to increase the effectiveness of the feedback and positive emotional experience [15]. Given this information, the impact of negative feedback on cognitive interventions should be investigated in order to improve the emotional experience, and subsequent mood outcomes, of CBM.

There are different ways to give feedback to participants in cognitive tasks. In this study, we present a new version of our previous CBM task, in which negative feedback is replaced by positive reinforcement of the desired response via gamification techniques that adapt game-like features to nongame tasks [16]. Gamification methods have been used in long and repetitive cognitive tasks to improve the engagement of participants [17,18]. The gamified cognitive bias modification (GCBM) uses a scoring system, instead of the “correct and incorrect” feedback, to change participant’s judgments of emotional faces, and potentially improve mood outcomes after the CBM training. The participants earn points for each correct answer but do not earn any points for the incorrect answers. They earn more points for “correct” answers to more ambiguous images that are harder to judge (ie, more ambiguous faces that are more difficult to judge).

In this study, we investigated whether replacing negative feedback with a gamified points feature delivers similar training effects to those seen in our previous studies [5-8]. The hypothesis is that a single session of GCBM would result in a positive change in the recognition of emotional facial expressions, similar to CBM training. We also, compared the effectiveness of a single session of GCBM training, CBM training, and CBM control conditions on immediate mood. Previous studies suggested that younger people benefited more from CBM training in terms of mood improvement [8]. Therefore, we recruited young adults between the ages of 18 and 30 years. We hypothesized that participants in the GCBM condition would have significantly higher immediate mood scores after training compared with the CBM group.

## Methods

### Overview

This study compared the effectiveness of a single session of CBM training, CBM (no training) control, and GCBM training on balance points and immediate mood. Preregistration information for this study is available on the Open Science Framework (OSF), where the experimental data can also be accessed [19]. There were deviations from the protocol as the CBM control condition was added post hoc. These deviations are explained in the Multimedia Appendix 1, but for clarity, we have presented the study as though all conditions were run concurrently (“Protocol Deviations: Adding a control condition post hoc” Multimedia Appendix 1). In addition, the development of the GCBM study, which tested its effectiveness on balance points with a smaller sample size, is provided in “Development and initial test of GCBM training” in Multimedia Appendix 1.

### Participants

The meta-analysis of CBM studies revealed an indirect effect size of g=0.23 for the effect of training on mood outcomes [9]. An a priori power analysis was conducted using G*Power [20]. We calculated a sample size of a total of 788 (n=394 in GCBM and n=394 CBM training conditions) would allow us to detect *d*=0.2 with 80% power. A further 120 participants were recruited for the control condition (refer to “Protocol Deviations: Sample size determination for the control group” in Multimedia Appendix 1 for this sample size determination). In total, we recruited 927 participants to allow for participants failing attention checks or not completing the study through Prolific Academic (Oxford University). The study was advertised to participants in the United Kingdom through Prolific. The study was run on Gorilla (Cauldron Science Ltd), a platform for creating web-based experimental tasks [21].

To be eligible for this study, participants had to be aged between 18 and 30 years, fluent in English, and have normal or corrected-to-normal vision. Participants were ineligible if they had consumed alcohol within the last 12 hours (self-report).

### Ethical Considerations

Ethics approval was obtained from the School of Psychological Science Research Ethics Committee at the University of Bristol (approval 15,735). The study was conducted according to the revised Declaration of Helsinki (2013) and the 1996 ICH (International Council for Harmonisation of Technical Requirements for Registration of Pharmaceuticals for Human Use) Guidelines for Good Clinical Practice E6 (R1).

At the beginning of the session, participants provided informed consent by checking a box after reading an information page. The consent form outlined that participation was voluntary, data would be anonymized and confidential, and participants could withdraw at any time. It also informed participants that anonymized data would be made publicly available as “open access” after the study, in line with best research practices. For full details of the consent form, please refer to the Multimedia Appendix 1. Participants were reimbursed £2.25 (US $2.84) via Prolific for their time (median completion time was 11, IQR 4.33 min).

### Study Design

This experimental study used a between-subjects design with 1 factor of intervention (CBM control, CBM training, and GCBM training) with immediate mood as the outcome. Participants were fully randomized to CBM and GCBM conditions with a 1:1 ratio by Gorilla randomizer. Participants were allocated to the control condition in a second phase of testing (refer to “protocol deviations” in Multimedia Appendix 1). Participants were blind to the condition to which they were assigned. Mood was self-assessed using the Immediate Mood Scaler (IMS) before and after training. The primary outcome is the difference in IMS scores between the CBM and GCBM groups.

### Materials

#### GCBM

**Figure 1.**
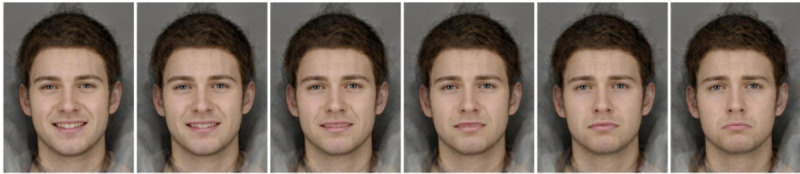
Examples of morphed faces used in the gamified cognitive bias modification intervention. The images depict unambiguously sad and happy expressions at the end points, with more ambiguous expressions in the center. These faces were presented to participants one at a time during the intervention.

The GCBM training used in this study was adapted from the CBM intervention used in our earlier work. The original CBM intervention involved repeated trials where the participant was briefly shown an image of a morphed emotional face and asked to decide whether the displayed face was happy or sad (refer to Figure 1 for example faces). These facial images (15 in total) were morphed between happy and sad emotions, taken from a continuum ranging from an unambiguously happy emotional face to an unambiguously sad emotional face, with ambiguous images toward the center of the continuum (Figure 1 illustrates an example of morphed happy and sad faces). Hence, each individual stimulus differed in the proportion of each emotion presented. The facial images are computer-generated faces and do not belong to any actual individuals [4].

**Figure 2.**
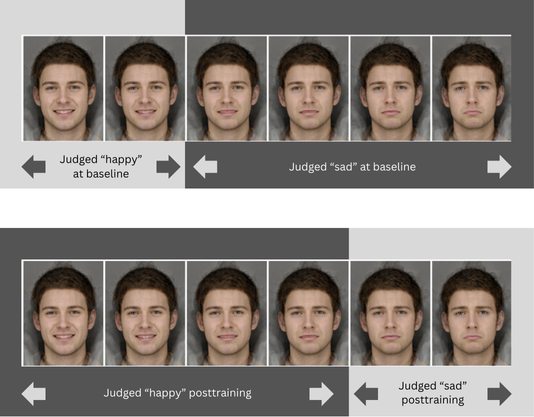
Example of intervention group responses to emotional facial expressions. The top image represents judgments at baseline, while the bottom image represents judgments after training.

The primary outcome was a “balance point,” which indicated the image at which a participant was equally likely to categorize the face as happy or sad. A lower balance point indicates a stronger negative emotion recognition bias toward ambiguous faces, as it reflects a tendency to classify more faces from the continuum as sad. Starting balance points were calculated for participants during the baseline block. In the training block, participants receive feedback on each trial, indicating whether their categorization was “correct” or “incorrect.” This feedback is tailored to each participant, based on their baseline balance point. For participants in the active training condition, feedback was designed to gradually shift their responses toward a more positive bias by categorizing more faces as happy. In the control condition, feedback aligned with their baseline balance point, meaning no shift in responses was expected. The balance point was calculated as the proportion of “happy” responses out of the total number of trials in the baseline or test block, multiplied by the total number of stimuli. For example, if a participant classified 30 out of 45 faces as happy during the baseline block, their baseline balance point would be (30÷45)×15=10. In the training condition, feedback would be given on the basis of a calculated target balance point of ([30÷45]×15+2=12; Figure 2 illustrates an example balance point change). Balance points were rounded to the nearest whole number for purposes of setting training feedback [4].

There were 4 blocks—baseline, 2 training blocks, and a test. The (identical) baseline and test blocks determined the participant’s pretraining and posttraining balance points. In a typical trial, a fixation cross was displayed for 600 ms, followed by a face stimulus for 200 ms. The baseline and test blocks consisted of 45 trials; the 15 facial stimuli were each presented 3 times in random order. All participants started with a baseline block to calculate their balance points. After that they continued to do the 2 training blocks, there were 31 trials, in which images 1-2 (unambiguously happy) and 14‐15 (unambiguously sad) were presented once, images 3-5 and 11-13 were presented twice, and images 6-10 were presented 3 times. This design was implemented because the most ambiguous images (6-10) were harder to classify, and most participants’ balance points tended to fall within this range, these images were presented more frequently to provide more training trails for the most difficult trials, at the critical level of ambiguity. In the training blocks, they received feedback based on their baseline balance point and the group they were in (training-control). After the training blocks, there was a test block in which the posttraining balance point was calculated. The experiment took roughly 10 minutes to complete.

**Figure 3.**
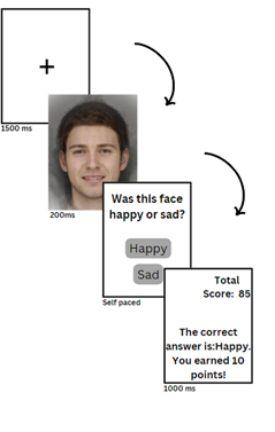
Example of a training block in the web-based gamified cognitive bias modification intervention.

The new GCBM intervention included the features mentioned above, but the method of feedback to participants was changed. In the CBM training, participants received positive or negative feedback as text (eg, “Correct! That face was happy” or “Incorrect! That face was Sad”). However, in the GCBM, participants earned points as a reward for correct answers but received no points for incorrect answers (Figure 3). In GCBM, more points were scored for correct answers closer to their baseline balance point so participants received a higher reward for correctly responding to more ambiguous expressions that were more difficult to classify (refer to “Scoring System for GCBM” in Multimedia Appendix 1 for scoring system details). Participants were able to see their total scores on the right upper corner of the screen throughout both training blocks. In the control condition, points were awarded based on a participant’s baseline balance point without any modification (ie, participants were rewarded for responding in the same way as they responded in the baseline block), and therefore no change in their balance point was expected.

GCBM also differs from CBM in terms of stimulus presentation duration and the use of visual noise masks. In GCBM, stimuli were presented for 200 ms (compared with 150 ms in CBM) to give participants more time to process facial expressions while minimizing the “flashing” effect. In addition, the visual noise mask used in CBM was removed, as participants in earlier trials reported it as unpleasant. These modifications were based on qualitative feedback from previous CBM studies to enhance participant experience.

#### IMS

IMS is a self-report questionnaire that measures well-being. It consists of 22 questions and responses are made on a 5-point scale (Multimedia Appendix 1).

#### Demographic Questions

Participants were asked about their ethnicity, gender, and age. The demographic questions were as follows:

- “Where are you from?” Participants were provided with a list of country options to select from.
- “What is your ethnicity?” Options included (1) Asian or Asian British; (2) Black, African, Caribbean, or Black British; (3) White; (4) Mixed or Multiple Ethnic Groups; (5) prefer not to say; and (6) other (please specify).
- “How old are you?” Participants answered using a text box.
- “What is your gender?” Options included (1) men, (2) women, (3) prefer not to say, and (4) other (please specify).

#### Attention Check

The participants were asked to type “seven” into a text box if they were still paying attention to the task after the IMS questionnaire.

### Procedure

At the beginning of the experiment, participants were presented with an information sheet and asked to complete a consent form via the web in which they confirmed eligibility against the criteria listed above. If participants confirmed eligibility and consent, they started the experiment by answering demographic questions. Next, they filled out IMS questionnaire for mood measurement. Next, the training was presented (either CBM control, CBM training, or GCBM training per the randomization). After the training, they completed the IMS questionnaire again and were presented with the feedback text box and attention check text box. Finally, they were provided with the debrief and researcher information, and reimbursed via their Prolific account.

### Statistical Analysis

We used box plots to identify and remove outliers (ie, data points that fall 1.5 times above or below the IQR). Data were assessed for normality using skewness and kurtosis statistics and were found to meet the assumptions of normality.

We conducted a linear regression analysis using SPSS 28 software (IBM Corp). This analysis compared posttraining balance points between the GCBM condition and other conditions (CBM training and CBM control), as well as all other pairwise combinations of conditions. We adjusted for baseline balance point and reported adjusted and unadjusted models.

To assess the effects of training on mood scores, we employed a mixed-design ANOVA. This analysis included a between-subjects factor with 3 levels—GCBM training, CBM training, and CBM control, and a within-subjects factor with 2 levels—pretraining and posttraining IMS scores. We looked at the interaction effect group and time, which assessed whether the change in mood scores from pretraining to posttraining varied across different groups.

We ran linear regression analysis using SPSS 28 software as a planned comparison to compare posttraining IMS scores in the CBM and GCBM conditions (the primary outcome in our protocol), and additionally all other pairwise combinations of conditions as post hoc tests. We adjusted for baseline mood, age, and gender, and reported adjusted and unadjusted models.

## Results

### Overview

**Figure 4.**
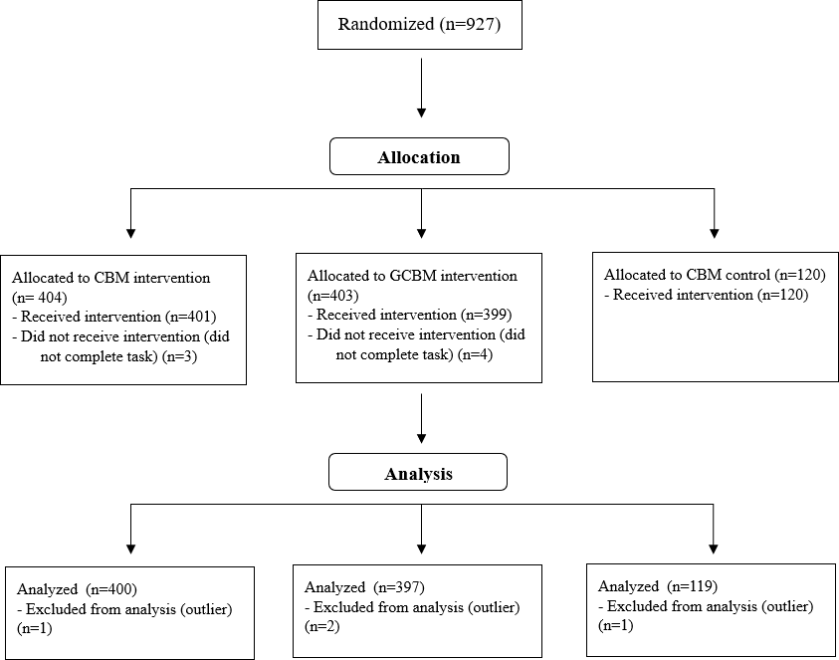
CONSORT (Consolidated Standards of Reporting Trials) flowchart. CBM: cognitive bias modification; GCBM: gamified cognitive bias modification.

We collected data from 927 participants (CBM=404, GCBM=403, and control=120) to account for potential exclusions due to failed attention checks and technical issues, and still reach our desired minimum sample size from our power calculation. Of the total, 7 participants did not complete the task (CBM=3, GCBM=4, and control=0). We removed 4 outliers that fell 1.5 times above or below the IQR (CBM=1, GCBM=2, and control=1) (refer to Figure 4 for CONSORT [Consolidated Standards of Reporting Trials] flowchart; Checklist 1). Of the remaining 916 participants (CBM=400, GCBM=397, and control=119), 55.1% (n=505) were women and 392 men; 19 participants did not state their gender. The mean age was 25.46 (SD 3.18) years. Out of 927, 76% (n=696) of the participants were White, 11.2% (n=103) were Asian, 6.6% (n=60) were Black, African, or Caribbean, 4.9% (n=45) were Mixed or from Multiple Ethnic Groups, and 1.3% (n=12) chose “other” as an option.

### Balance Points

**Figure 5.**
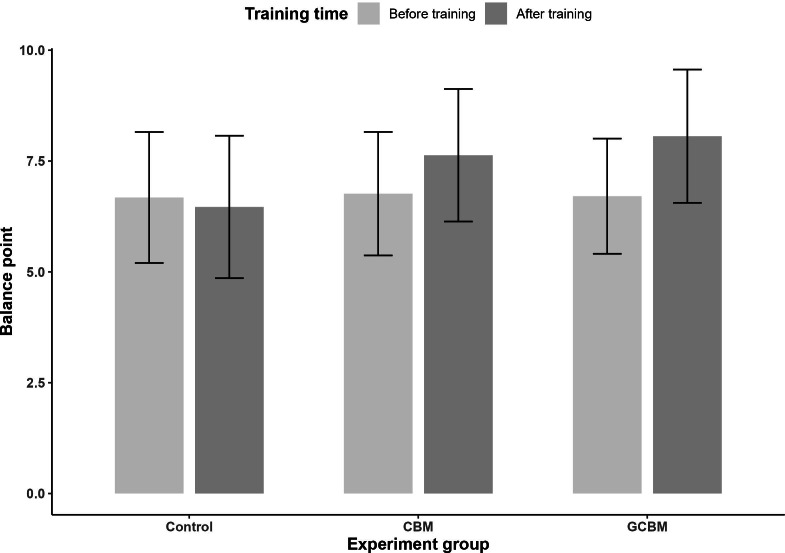
Graph for pre- and posttraining balance points for cognitive bias modification, gamified cognitive bias modification, and control conditions. Both cognitive bias modification and gamified cognitive bias modification training positively shifted participants’ balance points (higher balance points indicate participants categorized more faces as happy, suggesting a reduction in negative bias when interpreting ambiguous emotional facial expressions, and error bars represent SDs). CBM: cognitive bias modification; GCBM: gamified cognitive bias modification.

To assess the effect of training on emotion recognition bias, we examined participants’ balance points pre- and posttraining. Both CBM and GCBM training showed positive changes after training indicating that participants in both conditions categorized more ambiguous faces as “happy” after training while the control condition showed no meaningful change (Figure 5). On average, participants in CBM training showed a 0.87-point increase, while those in GCBM training demonstrated a 1.35-point increase.

To further evaluate the effect of cognitive bias training on balance points, we conducted a 3 (group: CBM, GCBM, and control)×2 (Time: pre-training and post-training) ANOVA with balance point as the dependent variable. The results showed evidence of an interaction effect between time (pre- and posttraining) and group (CBM, GCBM, and control), *F*_2913_=127.11, *P*<.001, partial *η*^2^=0.22.

We conducted exploratory, stratified linear regressions to compare posttraining balance points of CBM and GCBM training with each other and the control group. The results showed that both CBM and GCBM training groups had improved posttraining balance points compared with control group (B=−.55, 95% CI −0.64 to −0.45], *P*<.001; B=−1.57, 95% CI −1.76 to −1.38, *P*<.001, respectively) Also, there is evidence that GCBM training induced a larger training effect than CBM, (B=0.48, 95% CI 0.35-0.61, *P*<.001). These results are from the adjusted model for the pretraining balance point. Results observed in the unadjusted models were qualitatively the same in terms of direction and strength of evidence (Table S1 in Multimedia Appendix 1).

### Mood Outcomes

To assess the effect of training on immediate mood, we examined participants’ posttraining IMS scores. Participants in all conditions showed improved mood after the training. Control group had a 3.63-point improvement, CBM group had a 5.35-point improvement, and GCBM group had a 7.01-point improvement on average (Table 1). To evaluate the effect of cognitive bias training on immediate mood, a mixed method ANOVA with IMS as the outcome, time (pretraining or posttraining) as a within-subjects factor, and group (CBM, GCBM, and control) as a between-subjects factor was performed. The ANOVA results indicated that there is evidence for an interaction effect between time (pre- and posttraining IMS) and group (CBM, GCBM, and control), *F*_2913_=4.36, *P*=.01, partial *η*^2^=0.01.

**Table 1.** Descriptive statistics for pre- and posttraining Immediate Mood Scaler scores cognitive bias modification, gamified cognitive bias modification, and control conditions (higher Immediate Mood Scaler scores indicate better mood).

	CBM^a^		GCBM^b^		Control	
	Mean (SD)	n	Mean (SD)	n	Mean (SD)	n
Pretraining IMS score	96.39 (25.32)	400	98.12 (27.27)	397	95.50 (26.54)	119
Posttraining IMS score	101.74 (26.43)	400	105.13 (28.11)	397	99.13 (27.91)	119

a CBM: cognitive bias modification.

b GCBM: gamified cognitive bias modification.

To investigate this interaction effect, we ran stratified linear regressions to assess the effect of CBM, GCBM, and control groups on posttraining IMS scores as specified in our preregistration. The results indicated no substantial evidence that participants in the CBM training experienced improved immediate mood compared with the control group (B=−1.00, 95% CI −2.23 to 0.23, *P*=.11). However, participants in the GCBM training group reported improved immediate mood compared with the control group (B=−3.64, 95% CI −6.02 to −1.25, *P*=.003). In addition, there is weak evidence that GCBM leads to more positive changes in participants’ moods compared with CBM (B=1.69, 95% CI 0.02-3.37, *P*=.048; our primary prespecified outcome). These results are from adjusted model for pretraining IMS scores, age, and gender. Results observed in the unadjusted models were qualitatively the same in terms of direction and strength of evidence (Table S2 in Multimedia Appendix 1).

## Discussion

### Principal Findings

In this study, our objective was to assess the effectiveness of a new GCBM task to change judgments of emotional facial expressions and the immediate mood of participants. The results demonstrated that GCBM training positively changed the participants’ responses to emotional faces compared with control condition. Furthermore, findings suggest that GCBM is more effective than the CBM task at changing participants’ judgments of emotional expression. The results provided evidence that GCBM improved immediate mood more than the control and CBM groups.

Results showed that the difference between pre- and posttraining balance points was greater in both CBM and GCBM groups compared with control. This indicates that both training methods generated a positive shift in the responses to emotional facial expressions, in comparison with the control (no training) condition. Also, all 3 groups demonstrated an improvement in immediate mood after the training. However, participants in the GCBM group showed greater immediate mood improvement compared with those in the control and CBM conditions. There was no statistical evidence for a difference between the CBM and control groups in terms of immediate mood improvement. These results suggest that GCBM training is successful in both modifying responses to emotional facial expressions and enhancing participants’ immediate mood, above and beyond the (unusual, in our previous experience) positive effects of financial compensation for participation. The greater immediate mood improvement in the GCBM group, compared with both the CBM and control groups, suggests that the gamified aspect of the training may provide an additional benefit for improving mood beyond our original CBM intervention. This could imply that the engaging, game-like nature of GCBM might enhance participant motivation or make the training more impactful, leading to better mood outcomes.

### Comparison With the Previous Work

Participants in the GCBM group showed a greater positive shift in their responses to emotional faces compared with the CBM group. This result suggests that gamification of the task increased engagement with the cognitive target of the intervention. This outcome aligns with the rationale behind the CBM training which suggests that changing people’s perspective toward ambiguous facial expressions can improve their mood and potentially improve the symptoms of depression [4]. The results might suggest that greater improvement in emotion perception was associated with better improvement in immediate mood. On the other hand, greater improvement in immediate mood in the GCBM group might be solely the result of the absence of the negative effect of feedback in CBM as we predicted.

While some previous studies have shown mood improvement after multiple sessions of CBM training using the outlined method, most studies have not demonstrated significant evidence of improvement [5-8,22]. Results from a meta-analysis revealed that CBM has no overall positive effect on mood. However, the relationship between CBM and mood is mediated by the reduction in emotion recognition bias after training [9]. These results suggest that some therapeutic benefits of CBM may be masked or attenuated by the negative feedback in the training condition. GCBM was designed to address this issue by changing the delivery of feedback. Given that GCBM has been shown to reduce negative emotion recognition bias more effectively than CBM, it may be more effective when administered through multiple training sessions for longer-term mood improvement.

### Strengths and Limitations

One of the limitations of this study is that we only tested participants between the ages of 18 and 30 years old. This decision was based on previous research suggesting that younger participants tend to benefit more from this emotion recognition CBM task [8]. In this study, our primary objective was to gain a clear understanding of the effectiveness of gamifying this CBM task.

Second limitation of this study is that we did not examine the improvement of depressive symptoms; we only assessed the immediate mood of the participants. The IMS asks participants how they are feeling at the moment. However, previous CBM studies have used questionnaires that assess depressive symptoms. Although immediate mood can be an indicator of depressive symptoms [23], the effect of GCBM on depressive symptoms still needs further investigation.

Third limitation of the study is the absence of a control group where participants played a game unrelated to emotion recognition training. The only control group in this study was the CBM control condition, which is similar to CBM training but without the training component. This means we did not compare the impact of simply playing a game versus the impact of GCBM training on immediate mood. It is possible that GCBM influences immediate mood through the enjoyable aspects of playing a game.

Fourth limitation is that this study only assessed the effectiveness of a single session of GCBM on immediate mood. This approach was chosen to test whether GCBM effectively changes balance points similarly to CBM and to compare its mood-related effects with CBM. Economic considerations also influenced this decision, as detecting small effect sizes requires large samples, making multisession studies expensive. Before committing to a multisession study, we wanted to evaluate GCBM’s effectiveness on mood relative to CBM in a single-session format to determine its potential.

### Future Directions

Future studies should examine whether the greater improvement in immediate mood observed in GCBM is specifically driven by the absence of negative feedback or whether other factors, such as increased engagement or improved task design, contribute to its effectiveness. Investigating the specific mechanisms underlying these differences will help clarify the role of feedback in mood enhancement.

In addition, future research should assess the long-term effects of multiple GCBM training sessions on mood, as mood changes related to depression typically develop over an extended period [3].

Furthermore, future studies should compare GCBM with a similar game-based control condition to determine whether improvements in immediate mood are a result of the cognitive mechanisms targeted by the intervention or simply the enjoyment of playing a game.

Finally, further research is necessary to explore potential differences in how younger and older individuals respond to this CBM intervention, as age-related factors may influence its effectiveness.

### Conclusion

This study provided evidence that GCBM led to a more pronounced shift in participants’ responses to emotional faces after training compared with the CBM control and training conditions. Importantly, the immediate mood of participants in the GCBM group improved to a greater extent than in the CBM control and training conditions. These findings suggest that GCBM may be a promising and acceptable intervention for individuals experiencing mood disorders. Given the importance of developing remote and cost-effective interventions for depression, future studies should investigate the long-term effects of GCBM on depression symptoms and recruit participants diagnosed with depression, as they constitute the actual target group for GCBM.

## Supplementary material

10.2196/65103 Multimedia Appendix 1 Supplementary materials.

10.2196/65103 Checklist 1 CONSORT-eHEALTH checklist.

## Abbreviations

CBM: cognitive bias modification
CONSORT: Consolidated Standards of Reporting Trials
GCBM: gamified cognitive bias modification
ICH: International Council for Harmonisation of Technical Requirements for Registration of Pharmaceuticals for Human Use
IMS: Immediate Mood Scaler
OSF: Open Science Framework
