# Supplementary material for: Testing the Effectiveness of a Gamified Emotional Cognitive Bias Modification Task as an Intervention for Low Mood: Randomized Controlled Trial
Source: JMIR Serious Games. 2025 May 1;13:e65103. doi: 10.2196/65103 (PMC12061347; doi:10.2196/65103)

***S1: Protocol Deviations: Adding a control condition post hoc.***

In the protocol, we stated that we would compare the GCBM training and CBM training conditions only, without including any control groups, given the previous studies on CBM did not demonstrate pronounced mood enhancement after training (see Kuruoglu et al., 2024). Therefore, we collected data for CBM training and GCBM training conditions (400 participants for each group). We expected to find an improved mood in the GCBM group, while the mood in the CBM group would remain similar. Nonetheless, the results showed that both the GCBM and CBM groups’ moods improved after the training. Following these findings, our aim was to investigate whether these results stemmed solely from the training effects or were influenced by other factors, such as compensation provided for study participation. We hypothesised that the differences between pre-training and post-training mood scores can be attributed to the combined effects of compensation and GCBM/CBM training. To test this hypothesis, we collected data from a CBM training control group which was compensated but did not attempt to change emotion judgments to assess the effect of financial compensation alone on mood. Afterwards, we proceeded to compare the three groups: CBM training, GCBM training, and CBM control group, to allow us to estimate the role that compensation alone may play in our results. We hypothesized GCBM training group would show significantly higher post-training mood scores when compared to the control group.

***S2: Development and initial test of GCBM training***

Preregistration information for this study is available on the Open Science Framework, where the experimental data can also be accessed [1].

In this study aimed to investigate the effectiveness of a single session of GCBM on emotion perception and to assess whether the gamified version of the task would produce the same robust training effects on the interpretation of emotional expressions as the original CBM [2]. We hypothesised that there would be a positive change in the training group`s post-training balance point (BP, the measure of bias) compared to the control group.

***Method***

*Participants*

Previous studies [3,4] demonstrated a large (*Cohen’s d* = 1) change in balance point (i.e. a shift toward perception of happiness in ambiguous faces) in the intervention group compared to the control group. An a priori power analysis was conducted using G*Power [5] We calculated a sample size of a total of 54 (27 for each condition) to detect the *d* = 1 with 95% power.

We recruited 60 participants to allow for participants failing attention checks or not completing the study. We recruited participants through Prolific Academic and delivered the training using Gorilla, a platform for creating online experimental tasks [6]. Participants were reimbursed £3.75 for their time (median time is 10.19 minutes). Eligible participants were aged 18 years and over, fluent in English and had normal or corrected-to-normal vision. Participants were ineligible if they had consumed alcohol within the last 12 hours (self-report).

Ethics approval was obtained from the School of Psychological Science Research Ethics Committee at the University of Bristol (Approval Code: 14244). The study was conducted in accordance with the revised Declaration of Helsinki (2013) and the 1996 ICH Guidelines for Good Clinical Practice E6(R1).

*Study Design*

A between-subjects design with one factor (GCBM, no-training control) was used for this study. We tested the effectiveness of GCBM to train the positive bias in the emotion perception of participants (i.e. increasing ‘happy’ responses to ambiguous faces). Participants were randomised to control and training conditions with a 1:1 ratio. The balance point for emotion perception was measured before (baseline) and after training. The primary outcome was the emotion recognition bias, as assessed by the balance point (see below).

*Procedure*

At the beginning of the experiment, participants were presented with an information sheet and asked to complete an online consent form confirming eligibility against the criteria listed above. If participants confirmed eligibility and consent, they started the experiment by answering demographic questions. Next, the GCBM training was presented with participants being fully randomised to either the intervention or control group. After training, participants completed the Immediate Mood Scaler (IMS). Next, they filled out the feedback text box and attention check text box. Finally, they were debriefed and reimbursed via their Prolific account. The experiment took approximately 15 minutes.

*Materials*

This study used Gamified Cognitive Bias Modification (GCBM), the Immediate Mood Scaler (IMS), demographic questions, and an attention check, all of which are described in the main body of the paper.

*Statistical Analysis*

We conducted the statistical analyses using IBM SPSS Statistics for Windows, Version 28.0. We used box plots to identify and remove outliers (i.e., data points that fell 1.5 times above or below the interquartile range). There were no outliers to remove. Data were assessed for normality using skewness and kurtosis statistics and were found to meet the assumptions of normality.

We ran a linear regression analysis to compare the post-training balance points of the control and intervention groups. We adjusted for baseline balance point, age and gender, and reported adjusted and unadjusted models. The exploratory analysis investigated whether the intervention group had higher mood (IMS) scores after training compared to controls, again using regression with adjustment for baseline mood, age and gender.

***Results***

We collected data from 60 participants. Two participants were not eligible (consumed alcohol within 12 hours). Of the remaining 58 participants, 62.1% were women (32 women, 22 men, 4 did not state their gender). In the control condition, 58.6% were women and in the intervention condition, 65.5% were women. The mean age was 42.59 (SD = 13.38). 77.6% of the participants were White, 8.6% were Black/African/Caribbean, 6.9% were Mixed/Multiple Ethnic Groups, 3.4% were Asian, and 3.4% chose ‘other’ as an option. Figure 1 shows the average balance points for the control and intervention groups before and after the GCBM training.

**Figure S1:** Graph showing the balance points of control and intervention groups in the Gamified Cognitive Bias Modification (GCBM) intervention, measured pre- and post-training. Participants in the intervention group shifted their responses, while the control group showed no meaningful change.


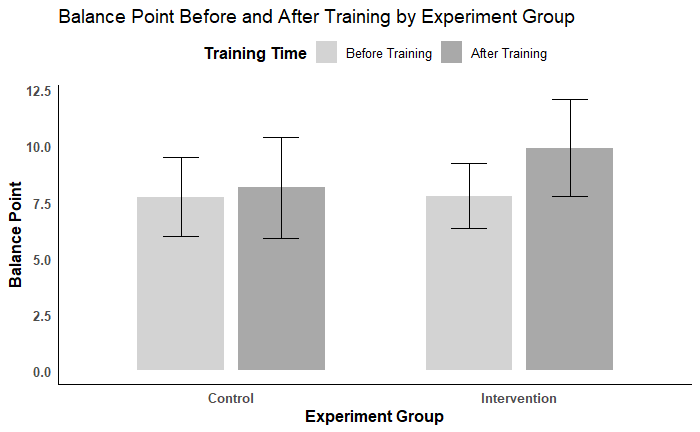


*Notes: Higher balance points indicate participants categorised more faces as happy, suggesting a reduction in negative bias when interpreting ambiguous emotional facial expressions. Error bars represent standard deviations*

The regression analysis results indicated that GCBM training led to positive changes in the balance point of participants (B = 1.77, 95% CI [0.61, 2.93], *P* = .004). This finding was robust to adjustment for baseline balance point, gender and age (Table 1). This suggests that participants in the intervention condition were more likely to classify ambiguous faces as 'happy' compared to controls after the training. On average, participants in the intervention condition showed an increase of 1.77 points in their balance point.

**Table S1:** Regression results for the effect of Gamified Cognitive Bias Modification (GCBM) on the post-training balance points.

| **Model** | |  | **Unstandardized Coefficients** | | | **Standardized Coefficients** | | |
| --- | --- | --- | --- | --- | --- | --- | --- | --- |
|  |  |  | **B** | **95% CI LL** | **95% CI UL** | **β** | | ***p*** |
|  | **1** | **Group (control and intervention)** | 1.77 | .61 | 2.93 | .38 | .004 | |
|  | **2** | **Group (control and intervention)** | 1.73 | 1.21 | 2.25 | .37 | <.001 | |
|  |  | **Pre-training balance point** | 1.23 | 1.07 | 1.40 | .83 | <.001 | |
|  | **3** | **Group (control and intervention)** | 1.73 | 1.24 | 2.22 | .37 | <.001 | |
|  |  | **Pre-training balance point** | 1.16 | .99 | 1.32 | .78 | <.001 | |
|  |  | **Gender** | -.20 | -.71 | .32 | -.04 | .445 | |
|  |  | **Age** | .03 | .01 | .05 | .17 | .004 | |

*Notes: Group is coded as control = 1 and intervention = 2. The outcome is the post-training balance point. Higher balance points indicate participants categorised more faces as happy which suggests less negative bias when interpreting ambiguous emotional facial expressions. Model 1 is unadjusted which is the effect of the different groups on the post-training balance point. Model 2 is adjusted for the pre-training balance point. Model 3 is additionally adjusted for age and gender.*

*Exploratory analysis*

We did not power this study based on the mood outcome, but we conducted exploratory analyses to examine the effect of group assignment (intervention vs. control) on mood outcomes. Results showed that group assignment did not predict significant differences in post-training mood scores (B = 1.40, 95% CI [-10.73, 13.52], *P* = .82).

***References***

1. Kuruoglu R. GCBM Study 1 Dataset. Available from: (https://osf.io/j6tr5/)

2. Penton-Voak IS, Bate H, Lewis G, Munafò MR. Effects of emotion perception training on mood in undergraduate students: randomised controlled trial. Br J Psychiatry 2012 Jul;201(1):71–72. doi: 10.1192/bjp.bp.111.107086

3. Rawdon C, Murphy D, Motyer G, Munafò MR, Penton-Voak I, Fitzgerald A. An investigation of emotion recognition training to reduce symptoms of social anxiety in adolescence. Psychiatry Research 2018 May;263:257–267. doi: 10.1016/j.psychres.2018.02.023

4. Suddell S, Müller-Glodde M, Lumsden J, Looi CY, Granger K, Barnett JH, Robinson OJ, Munafò MR, Penton-Voak IS. Emotional bias training as a treatment for anxiety and depression: evidence from experimental medicine studies in healthy and medicated samples. Psychol Med 2023 Feb;53(3):696–705. doi: 10.1017/S0033291721002014

5. Faul F, Erdfelder E, Lang A-G, Buchner A. G*Power 3: A flexible statistical power analysis program for the social, behavioral, and biomedical sciences. Behavior Research Methods 2007 May;39(2):175–191. doi: 10.3758/BF03193146

6. Anwyl-Irvine AL, Massonnié J, Flitton A, Kirkham N, Evershed JK. Gorilla in our midst: An online behavioral experiment builder. Behav Res 2020 Feb;52(1):388–407. doi: 10.3758/s13428-019-01237-x

***S3: Protocol Deviations:*** ***Sample size determination for the control group***

We conducted an additional power calculation for the control group based on the effect sizes of the differences between the pre and post-training Immediate Mood Scaler (IMS) scores. The effect sizes are moderate for both groups, with d = 0.58 for GCBM and d = 0.45 for CBM. An a priori power analysis was performed using G*Power (Faul, Erdfelder, Lang, & Buchner, 2007). Our calculations indicated that a sample size of 115 participants in the control group would provide us with the ability to detect an effect size of d = 0.45 with a power of 99%. We recruited 120 participants to allow for participants failing attention checks or not completing the study. We ran the CBM training control condition on this group of 120 participants to investigate whether the differences observed between pre and post-training mood scores are indeed attributable to the effects of CBM and GCBM training.

***S4: Scoring System for GCBM***

The balance point (for details, see Study 1) was calculated in the baseline block for each participant before training and used as their baseline score. In the GCBM training condition, participants were trained to shift their balance points by 2 points, enabling them to categorize 2 more faces as happy. After the baseline block participants started training blocks and were asked to choose happy or sad for each face. Incorrect answers for these faces resulted in zero points, while correct answers earned points based on the ambiguity of the faces. Faces closest to the participant’s balance point (the most ambiguous one above and one below) were worth 15 points. The scoring equation used was: Score = Max Score – |Stimuli Level – Rounded Balance Point| where the maximum score was 15, stimuli level ranged from 0 to 15 (0 for happy, 15 for sad, with morphed faces in between), and the Rounded Balance Point was the participant's balance point rounded to the nearest integer. For example, if a participant in the training condition gets a baseline balance point is 7 we train them to increase their balance point to 9. In this case, when they see the Stimuli Level 8 and find the correct answer they receive 14 points. This method allowed each participant to earn personalized points based on their specific balance point.

**Table S1:** Regression results comparing Cognitive Bias Modification (CBM), Gamified Cognitive Bias Modification (GCBM), and control conditions balance points.

| **Model** | | **Unstandardized Coefficients** | | | **Standardized Coefficients** | |  |
| --- | --- | --- | --- | --- | --- | --- | --- |
|  |  | **B** | **95% CI LL** | **95% CI UL** | **β** | ***p*** |  |
| **1** | **Group (CBM, control)** | -.55 | -.64 | -.45 | -.29 | <.001 |  |
|  | **Pre-training BP** | .84 | .78 | .90 | .74 | <.001 |  |
| **2** | **Group (GCBM, control)** | -1.57 | -1.76 | -1.38 | -.40 | <.001 |  |
|  | **Pre-training BP** | .91 | .85 | .97 | .73 | <.001 |  |
| **3** | **Group (CBM, GCBM)** | .48 | .35 | .61 | .16 | <.001 |  |
|  | **Pre-training BP** | .87 | .82 | .92 | .78 | <.001 |  |
| *Notes: Group is coded as CBM = 1, GCBM = 2, and control = 3. The outcome is the post-training balance point. Higher balance points indicate participants categorised more faces as happy which suggests less negative bias when interpreting ambiguous emotional facial expressions. Model 1: Comparison of the effect of CBM and CBM control on balance points. Model 2: Comparison of the effect of GCBM and CBM control on balance points. Model 3: Comparison of the effect of CBM and GCBM on balance points.* | | | | | | | |

| **Table S2:** Regression results comparing Cognitive Bias Modification (CBM), Gamified Cognitive Bias Modification (GCBM), and control conditions on mood outcome Immediate Mood Scaler (IMS). |
| --- |
| \| **Model** \| \| **Unstandardized Coefficients** \| \| \| **Standardized Coefficients** \| \| \| \| \| --- \| --- \| --- \| --- \| --- \| --- \| --- \| --- \| --- \| \| **B** \| **95% CI LL** \| **95% CI UL** \| **β** \| \| ***p*** \| \| \| **1** \| **Group (CBM, control)** \| -.97 \| -2.21 \| .27 \| -.03 \| \| .125 \| \| \| **Pre-training IMS score** \| .93 \| .89 \| .97 \| .90 \| \| <.001 \| \| \| **2** \| **Group (CBM, control)** \| -1.00 \| -2.23 \| .23 \| -.03 \| \| .112 \| \| \| **Pre-training IMS score** \| .93 \| .89 \| .97 \| .89 \| \| <.001 \| \| \| **Age** \| -.25 \| -.59 \| .08 \| -.03 \| \| .135 \| \| \| **Gender** \| 2.36 \| .25 \| 4.46 \| .04 \| \| .028 \| \| \| **3** \| **Group (GCBM, control)** \| -3.64 \| -6.02 \| -1.25 \| -.05 \| \| .003 \| \| \| **Pre-training IMS score** \| .94 \| .91 \| .98 \| .91 \| \| <.001 \| \| \| **4** \| **Group (GCBM, control)** \| -3.64 \| -6.02 \| -1.25 \| -.05 \| \| .003 \| \| \| **Pre-training IMS score** \| .94 \| .91 \| .98 \| .91 \| \| <.001 \| \| \| **Age** \| -.03 \| -.34 \| .28 \| .00 \| \| .860 \| \| \| **Gender** \| .21 \| -1.82 \| 2.24 \| .00 \| \| .838 \| \| \| **5** \| **Group (CBM, GCBM)** \| 1.73 \| .05 \| 3.41 \| .03 \| \| .044 \| \| \| **Pre-training IMS score** \| .93 \| .90 \| .96 \| .90 \| \| <.001 \| \| \| **6** \| **Group (CBM, GCBM)** \| 1.69 \| .02 \| 3.37 \| .03 \| .048 \| \| \| **Pre-training IMS score** \| .93 \| .90 \| .96 \| .90 \| <.001 \| \| \| **Age** \| -.19 \| -.45 \| .07 \| -.02 \| .147 \| \| \| **Gender** \| 1.60 \| -.09 \| 3.29 \| .03 \| .064 \| \| |

*Notes: Group is coded as CBM = 1, GCBM = 2, and control = 3. Gender coded as Women =1 Men =2. The outcome is the post-training IMS score. Higher IMS scores indicate better mood. Model 1: Comparison of the effect of CBM and control on mood adjusted for pre-training IMS. Model 2: Comparison of the effect of CBM and control on mood adjusted for age and gender as well Model 3: Comparison of the effect of GCBM and control on mood adjusted for pre-training IMS. Model 4: Comparison of the effect of GCBM and control on mood additionally adjusted for age and gender. Model 5: Comparison of the effect of CBM and GCBM on mood, adjusted for pre-training IMS. Model 6: Comparison of the effect of CBM and GCBM on mood, adjusted for age and gender as well.*

***S5: Consent Form***

Please take a moment to read the text below to understand how **your data** will be used.

**What will happen to my data?**

Your involvement in the study will remain confidential. This information will only be available to research staff and national bodies which monitor whether research studies are conducted properly. Your study data will be anonymised. This means that it will be given an identification number and any identifying information about you will be removed. Therefore, it will not be possible to identify you by name from any aspect of documentation or reporting for this research study. At the end of the study, your data will be made “Open Access”. This means that it will be stored in an online database so that it is publicly available.

**What is open access?**

Open access means that data are made available, free of charge, to anyone interested in the research, or who wishes to conduct their own analysis of the data. We will therefore have no control over how these data are used. However, all data will be anonymised before it is made available and therefore there will be no way to identify you from the research data.

**Why open access?**

Open access of research data and findings is considered best research practice and is a requirement of many funding bodies and journals. As a large proportion of research is publicly funded, the outcomes of the research should be made publicly available. Sharing data helps to maximise the impact of investment through wider use, and encourages new avenues of research.

Thank you for reading. If at any point of the study you feel uncomfortable and want to withdraw from the study, please feel free to exit the tab (your data will not be kept if you do so). If you want to withdraw your data after completion, please let me know through email. However, deletion of your data after completion of the study is not always possible as it is difficult to identify your data because it is anonymised.

For any enquiries, please email me at ey21541@bristol.ac.uk

**Check the box below to confirm your consent and begin the study.**

By checking this box, I understand that after the study the data will be made “open data”. I understand that this means the anonymised data will be publicly available and may be used for purposes not related to this study, and it will not be possible to identify me from these data. I confirm that I have been given sufficient information about the study and enough time to seek clarification from the researcher.

***S6: Participant Information Sheet***

**Title of the study: TESTING THE EFFECTIVENESS OF A GAMIFIED VERSION OF EMOTION RECOGNITION TRAINING**

Thank you for taking the time to participate in this study. Before you decide you need to understand why the research is being done and what it would involve for you. Please take time to read the following information carefully. Take time to decide whether or not to take part.

**What is this study about?**

I am Rumeysa Kuruoglu 2^nd^ year PhD student in Psychological Sciences, at the University of Bristol. This study has been approved by the University of Bristol Research Ethics Committee. If you agree to take part in this research I will ask you to complete a task and fill out some questionnaires. This study aims to understand how different people perceive emotional faces. You will start by filling in a demographic survey, followed by a face perception task and some questions. All of this will take roughly 15 minutes.

**Participation to study**

Your participation in this research is completely voluntary. If at any point you wish to no longer take part in the research you have the right to withdraw at any time and there will be no pressure to stay.

All the information you give **will be anonymous and confidential** and only used for the purposes of this research.

Thank you very much for your time and help!

***S7: Debrief***

Thank you for taking the time to participate! Please read through this page - it contains information about the nature of the study.

**What is this study about?**

Depression is associated with a negative bias in the perception of facial emotional expressions. expressions (e.g. Penton-Voak, Bate, Lewis, & Munafò, 2012; Bourke, Douglas, & Porter, 2010). This negative bias has been proposed to play an important role in the onset and maintenance of depression (e.g. Harmer, Goodwin, & Cowen, 2009; Persad & Polivy, 1993). In this experiment, we want to change negative biases in the perception of emotional facial expressions. This experiment is comparing the original and the new version of the CBM. task (Penton-Voak et al., 2012). **In this study, we want to recruit a typical population to see if this task is working in the way we hope, with hopes of developing an intervention for use with clinical populations in the future.**

**Please email me at ey21541@bristol.ac.uk for any queries.**

Interested in the results of this study? If you'd like to be forwarded the outcome of this study once it is ready, please provide your email below. Otherwise, leave it blank.

***S8: Demographics Questions***


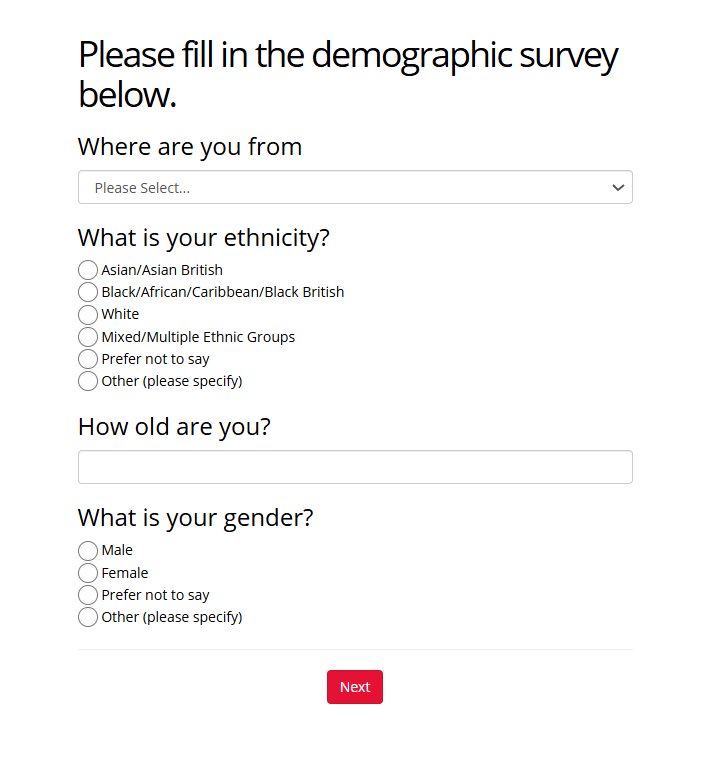


***S9: Immediate Mood Scaler***


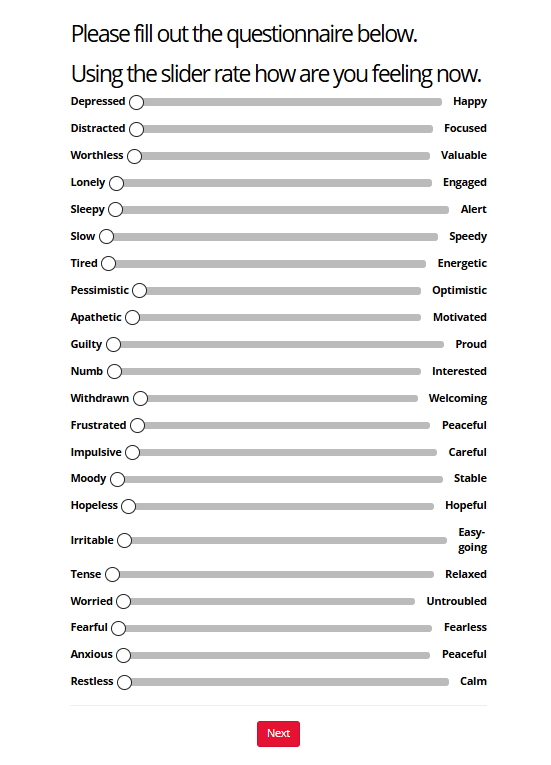

Supplement: Multimedia Appendix 1 [file games-v13-e65103-s001.docx]
